# The impact of radiologists’ expertise on screen result decisions in CT lung cancer screening

**DOI:** 10.1186/1470-7330-14-S1-P19

**Published:** 2014-10-09

**Authors:** MA Heuvelmans, M Oudkerk, PA de Jong, WPTM Mali, HJM Groen, R Vliegenthart

**Affiliations:** 1 Center for Medical Imaging – North East Netherlands, Department of Radiology, University of Groningen, University Medical Center Groningen, Groningen, Netherlands; 2 Department of Radiology, University Medical Center Utrecht, Utrecht, Netherlands; 3 Department of Pulmonology, University of Groningen, University Medical Center Groningen, Groningen, Netherlands

## Aim

To evaluate the impact of radiological expertise on screen result decisions made in a CT lung cancer screening trial.

## Methods

In the Dutch-Belgian randomized lung cancer screening trial (NELSON), the baseline CT screen result was based on the lung nodule with largest volume. According to the protocol, nodule volume<50mm3, 50-500mm3 and >500mm3 led to a negative, indeterminate and positive screen result, respectively. The protocol, however, allowed radiologists to manually adjust screen result in case of high suspicion of benign or malignant nodule nature. In this study, all participants whose baseline CT result was based on a solid nodule were included. Adjustments by radiologists at baseline were evaluated. Histology was the reference for diagnosis, or, to confirm benignity, stability on subsequent CT scans.

## Results

3,268 participants (2,759 male, median age 58.0-years) were included. In 189 participants (5.8%) the initial baseline screen result was adjusted by the radiologist. Adjustment was downwards from positive or indeterminate to negative in two and 118 participants, respectively, and from positive to indeterminate in 64 participants. None of these nodules turned out malignant. In five participants (2.6%) the screen result was adjusted upwards from negative to indeterminate (N=1) or indeterminate to positive (N=4); two nodules (40%) were malignant.

## Conclusion

In about one-in-twenty cases of baseline lung cancer screening, nodules were reclassified by the radiologist (97.4% downwards), leading to reduction of false-positive and false-negative screen results.

